# Lifetime Exposure to Endogenous Estradiol and Markers of Dementia Risk: Associations with Later Life Cognitive, Behavioral, and Functional Complaints

**DOI:** 10.3390/diagnostics16081146

**Published:** 2026-04-12

**Authors:** Jasper F. E. Crockford, Dylan X. Guan, Maryam Ghahremani, Clive Ballard, Byron Creese, Anne Corbett, Ellie Pickering, Adam Bloomfield, Pamela Roach, Cindy K. Barha, Eric E. Smith, Zahinoor Ismail

**Affiliations:** 1 Faculty of Graduate Studies, University of Calgary, Calgary, AB T2N 4N1, Canada; 2 Cumming School of Medicine, University of Calgary, Calgary, AB T2N 4N1, Canada; 3 Hotchkiss Brain Institute, University of Calgary, Calgary, AB T2N 4N1, Canada; 4 Department of Psychiatry, University of Calgary, Calgary, AB T2N 4N1, Canada; 5 Clinical and Biomedical Sciences, Faculty of Health and Sciences, University of Exeter, Exeter EX4 4PY, UK; 6 Department of Community Health Sciences, University of Calgary, Calgary, AB T2N 4N1, Canada; 7 Department of Family Medicine, University of Calgary, Calgary, AB T2N 4N1, Canada; 8 Department of Kinesiology, University of Calgary, Calgary, AB T2N 1N4, Canada; 9 Department of Clinical Neurosciences, University of Calgary, Calgary, AB T2N 4N1, Canada; 10 O’Brien Institute for Public Health, University of Calgary, Calgary, AB T2N 4N1, Canada; 11 Department of Pathology and Laboratory Medicine, University of Calgary, Calgary, AB T2N 4N1, Canada

**Keywords:** estradiol, cognition, behavior, function, dementia risk

## Abstract

**Background/Objectives**: Longer lifetime exposure to endogenous estradiol (L_EE2_) has been associated with lower risk of age-related cognitive decline and dementia. Complementary to cognitive decline, behavioral and functional decline are also predictive of dementia risk; however, the association between L_EE2_ and these domains is underexplored. We investigated whether L_EE2_ is correlated with later-life changes in behavior and function. **Methods**: Baseline data from 1156 females enrolled in the CAN-PROTECT study were analyzed. L_EE2_ was estimated based on the length of the reproductive period (menopause age–menarche age) plus years pregnant and scaled in 5-year increments. Objective cognition was measured using the CAN-PROTECT neuropsychological battery, while subjective cognition, behavior, and function were measured using the Revised Everyday Cognition (ECog-II) scale, Mild Behavioral Impairment Checklist (MBI-C), and Standard Assessment of Global Everyday Activities (SAGEA) scale, respectively. Linear regressions modeled the association between L_EE2_ and neuropsychological performance. Three separate negative binomial regression models examined the association between L_EE2_ and ECog-II, MBI-C, and SAGEA total scores. All models adjusted for menopause hormone therapy, menopause type, age at first childbirth, body mass index, age, education, and ethnocultural background. **Results:** Each five-year increase in L_EE2_ was associated with a lower MBI-C score (count ratio [CR] = 0.89, 95% CI [0.82, 0.97]) and lower SAGEA score (CR = 0.91, 95% CI [0.84, 0.98]). L_EE2_ was not significantly associated with any objective or subjective cognitive measures. **Conclusions**: Longer L_EE2_ may associate with lower severity of later-life behavioral and functional symptoms in older women.

## 1. Introduction

Alzheimer disease (AD) is the most common cause of dementia, accounting for approximately 60–70% of all cases worldwide [1]. An estimated 55 million people currently live with dementia, a number projected to nearly triple by 2050 as populations age [2]. Notably, females represent nearly two-thirds of individuals with AD [3], which cannot be fully explained by greater longevity alone [4]. This sex disparity underscores the importance of understanding biological and life course factors that may confer susceptibility or resilience to AD in females.

Although recent advances in disease-modifying therapies have shown promise for some patients, their impact on clinical outcomes remains modest [5,6]. This has increased the importance of identifying early risk markers and modifiable factors that precede the onset of AD. One such avenue involves understanding how hormonal exposures across the female lifespan correlate with later-life brain health. From menarche (first menstruation) through the reproductive years (including pregnancy) to menopause (permanent cessation of menses), females experience dynamic fluctuations in reproductive hormones, particularly estradiol (E2), the most abundant and potent form of circulating estrogen. E2 exerts widespread neuroprotective effects [7] including supporting vascular integrity [8], promoting synaptic plasticity [9], modulating neurotransmitter [10] and inflammatory systems [11], and facilitating the clearance of amyloid-beta and phosphorylated tau [7], hallmark proteinopathies of AD. Consequently, variations in E2 exposure have been proposed as one mechanism linking female reproductive aging to later-life brain health and AD risk.

Beyond post-menopausal decline in E2, lifetime exposure to endogenous estradiol (L_EE2_) may also play a role in shaping brain aging trajectories. L_EE2_ duration can be approximated by the interval of time between menarche and menopause [12], with additional years of pregnancy contributing to exposure given the markedly elevated E2 levels during gestation [13,14,15]. Shorter L_EE2_ has been associated with poorer performance on cognitive tasks (including poorer delayed memory recall [12] and global cognition [15]), and greater risk of white matter hyperintensity burden [8,14] and dementia [16] in later life. Conversely, longer L_EE2_ may confer neuroprotective benefits through prolonged exposure to the supportive effects of E2 on neural [15], vascular [14], and inflammatory [17] processes.

While emerging evidence links L_EE2_ to cognitive outcomes, few studies have examined the association of L_EE2_ with other early markers of neurodegenerative disease, such as neuropsychiatric symptoms (NPS) and functional decline. Both NPS and functional decline are predictive of dementia risk and progression and may precede overt cognitive impairment [18]. When NPS are later-life emergent and persistent, they are classified as mild behavioral impairment (MBI) [19], which has been associated with neurodegeneration [20] and AD cerebrospinal [21] and plasma [22,23] fluid biomarkers, even after accounting for cognitive status. Similarly, subtle functional difficulties (e.g., managing medications, preparing meals, travelling, and managing finances) may also signal early-stage neurodegenerative changes [24,25]; recent work has linked mild functional impairment in cognitively unimpaired older adults to both incident dementia and AD biomarkers [26,27]. Although no studies to date have directly examined L_EE2_ in relation to MBI or functional decline, premature menopause has been linked to greater depressive symptoms [28] and poorer physical function [29] in later life, suggesting that duration of E2 exposure may influence behavioral and functional outcomes. Thus, extending investigations of L_EE2_ beyond cognition may strengthen early identification of females at elevated risk of neurodegeneration.

In addition to endogenous E2 exposure, menopause hormone therapy (MHT) may also influence brain aging and dementia risk, though findings remain inconsistent [30]. Variability in MHT timing of initiation, duration of use, and formulation of E2, as well as individual health characteristics may contribute to these inconsistencies [31]. These findings underscore the need to account for MHT use in L_EE2_ models when evaluating their effects on brain aging.

The present study examined the associations between L_EE2_, global measures of cognition, behavior, and function in a sample of postmenopausal females. We hypothesized that longer L_EE2_ would associate with lower severity of cognitive, behavioral, and functional symptoms in later life.

## 2. Materials and Methods

### 2.1. Study Design

Data were drawn from the Canadian Platform for Research Online to Investigate Health, Quality of Life, Cognition, Behaviour, Function, and Caregiving in Aging (CAN-PROTECT) [32], a digital epidemiology platform longitudinally investigating risk and resilience in brain aging [33,34,35]. To be eligible for CAN-PROTECT, participants must be aged 18 years or older, reside in Canada, be dementia-free at enrollment, and have access to an internet-connected computer or tablet. Participants complete annual mandatory neuropsychological assessments and demographic questions, as well as optional assessments of cognition, behavior, function, quality of life, medical and psychiatric history, and lifestyle. Participants who reported female sex at birth were also invited to complete a fertility and menopause questionnaire. All participants provide informed consent electronically at registration. The CAN-PROTECT study was approved by the Conjoint Health Research Ethics Board at the University of Calgary, with recruitment ongoing since 8 March 2023.

### 2.2. Participants

**Figure 1:**
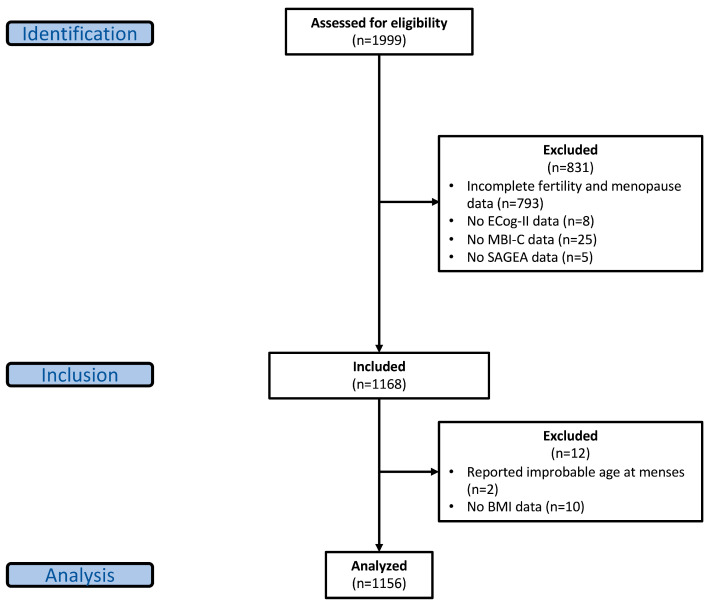
Flowchart of analyzed CAN-PROTECT participants. Abbreviations: ECog-II, Revised Everyday Cognition scale; MBI-C, Mild Behavioral Impairment Checklist; SAGEA, Standard Assessment of Global Everyday Activities scale; BMI, body mass index.

Baseline demographic, cognitive, behavioral, functional, and reproductive health data were available for 1999 participants. Inclusion in the analysis required complete data on neuropsychological battery tests, subjective cognitive, behavioral, and functional measures, reported female sex at birth, self-reported postmenopausal status, and age at menarche and menopause. Of these, 1168 met inclusion criteria; however, two participants were excluded due to biologically implausible values for age at menarche (i.e., menarche after 40 years of age) and an additional 10 participants were excluded due to no body mass index (BMI) data. The final analytic sample comprised 1156 participants (Figure 1).

### 2.3. Measures

#### 2.3.1. Lifetime Exposure to Endogenous E2

Lifetime exposure to endogenous E2 (L_EE2_) was estimated by using self-reported reproductive history from the fertility and menopause questionnaire. Specifically, reproductive span was calculated as the difference between reported age at menopause and age at menarche [12]. To account for elevated E2 during pregnancy [13], an additional 0.75 years (equivalent to nine months) was added for each reported biological child, an approach previously used to examine L_EE2_ and white matter hyperintensities [14]. This approach represents a simplified proxy of cumulative endogenous E2 and does not capture variability in E2 levels across pregnancies or postpartum factors, which were not available in CAN-PROTECT.

For regression analyses, L_EE2_ was scaled in five-year increments to improve interpretability of effect estimates. Data on miscarriages and preterm births are not collected in CAN-PROTECT and therefore were not included in L_EE2_ estimates.

#### 2.3.2. MHT

Ever use of MHT was self-reported and included unopposed estrogen (e.g., E2, conjugated estrogens) and opposed estrogen (e.g., combination estrogen and progestin) forms. Data on MHT use indicated for reasons other than menopause symptom treatment are not collected in CAN-PROTECT, and thus were not identified as MHT users. MHT use was categorized as either never or ever use.

#### 2.3.3. L_EE2_-Related Variables

Menopause type was included as a covariate and categorized as spontaneous, surgical, or due to other reasons. CAN-PROTECT captures menopause resulting from oophorectomy or hysterectomy as a single category, precluding distinction between these two surgical procedures.

Age at first childbirth was measured continuously and included as a covariate, given prior evidence linking later age at first childbirth to lower AD risk [36]. BMI, a factor linked to endogenous E2 levels [37], was also included and calculated from self-reported height and weight.

#### 2.3.4. Neuropsychological Performance

Objective measures of cognition were assessed using the validated CAN-PROTECT neuropsychological battery [38], which is comprised of six assessments including: Trail Making B, Switching Stroop, Self-Ordered Search, Paired Associate Learning, Verbal Reasoning, and Digit Span.

Raw test scores were extracted across the six domains and winsorized at the first and 99th percentiles to minimize outlier effects. Each task was then standardized into a z-score based on the sample distribution, with time-based measures reverse-coded so that higher z-scores consistently reflected better performance. Domain composite scores were calculated as the mean of all non-missing standardized task scores within each domain, and a global composite neuropsychological score was derived as the mean of all available task z-scores per participant.

#### 2.3.5. Everyday Cognition Scale

Cognition was also assessed using the Revised Everyday Cognition (ECog-II) scale [39], a 41-item measure developed to detect subtle cognitive impairment in populations at risk for dementia. Items cover memory, language, visual-spatial and perceptual, planning, organizational, and executive function, with participants rating perceived change over the past ten years on a scale from 0–3 (0 = no change, 1 = occasionally worse, 2 = consistently a little worse, 3 = much worse). Total ECog-II scores were calculated by summing all item severity scores, with higher scores indicating greater perceived impairment.

#### 2.3.6. Mild Behavioral Impairment Checklist

Behavioral symptoms were assessed using the Mild Behavioral Impairment Checklist (MBI-C) [40], a 34-item measure designed to capture late-life onset, persistent (≥6 months), and impactful behavioral changes not explained by established psychiatric conditions or other diagnoses. The MBI-C demonstrates validity as a marker of clinical and biological AD risk [19,20,21,41]. The MBI-C covers five domains: decreased motivation, affective dysregulation, impulse dyscontrol, social inappropriateness, and abnormal perception and thought content. Items are rated from 0–3 (0 = no symptom, 1 = mild symptom, 2 = moderate symptom, 3 = severe symptom). Total MBI-C scores were calculated by summing all item severity scores, with higher total scores indicating greater behavioral disturbance.

#### 2.3.7. Standard Assessment of Global Everyday Activities Scale

Functional ability was measured using the Standard Assessment of Global Everyday Activities (SAGEA) scale [42], a 15-item, multidomain measure of functional capacity over the past month. The SAGEA has demonstrated validity as a dementia screening tool [24,25,43]. The SAGEA assesses instrumental activities of daily living, basic activities of daily living, cognition, social participation, and mobility. Items are rated on a 0–3 scale (0 = no impairment, 1 = mild impairment, 2 = moderation impairment, 3 = severe impairment), with higher scores indicating greater functional impairment. For four activities, participants are additionally asked whether they require assistance from another person; endorsement of assistance contributes additional points, up to a maximum of 3 points per item. Total SAGEA scores were calculated as the sum of all domain scores.

### 2.4. Statistical Analysis

Participant demographics and outcome variables were summarized using descriptive statistics (count, percentages, mean, and standard deviations). Distributions of outcome variables were visually inspected using histograms and Q-Q plots and further assessed for skewness, kurtosis, and dispersion to guide model selection. L_EE2_ was modeled as a continuous exposure variable, scaled in five-year increments to enhance interpretability of effect estimates.

The relationship between L_EE2_ (exposure) and neuropsychological performance (global and domain) was modeled using linear regressions. Comparatively, the associations of L_EE2_ (exposure) and the outcome variables, ECog-II, MBI-C, and SAGEA total scores, were modeled as overdispersed count outcomes using separate negative binomial regressions. Exponentiated coefficients from these models (using a log link) are presented as count ratios (CRs), representing the proportional change in the expected count of the outcome per one-unit increase in the predictor. This terminology was used instead of incidence rate ratios because no offset term—used to account for differing exposure times or observation periods—was included; thus, the models estimated counts rather than rates.

All models were adjusted for MHT use (never versus ever), menopause type (spontaneous, surgical, or other), age at first childbirth, BMI, age, years of education, and ethnocultural (European versus no European) background.

## 3. Results

A total of 1156 females were included in the analyses. Participants had a mean age of 63.9 ± 7.4 years and reported a mean number of 15.8 ± 4.5 years of education. Most participants (85.1%) reported at least some European ethnocultural background and a BMI average of 24.3± 4.8 (Table 1).

The mean age at menarche was 12.7 ± 1.5 years, while menopause onset occurred at a mean age of 49.7 ± 5.8 years. Most participants experienced spontaneous menopause (76.0%). While 23.2% of the sample reported having no biological children, the majority reported at least one, with an average age at first childbirth of 27.3 ± 5.2 years and an average of 1.3 ± 0.9 total years pregnant. Combining reproductive period with years pregnant, the mean years of L_EE2_ was 38.2 ± 5.9. Approximately 35.1% reported MHT use at some point.

On average, participants reported a global neuropsychological performance z-score of 0.0 ± 0.5, ECog-II severity score of 12.5 ± 11.8, MBI-C severity score of 5.9 ± 7.7, and SAGEA severity score of 2.7 ± 3.5.

### 3.1. L_EE2_ and Cognition

**Figure 2:**
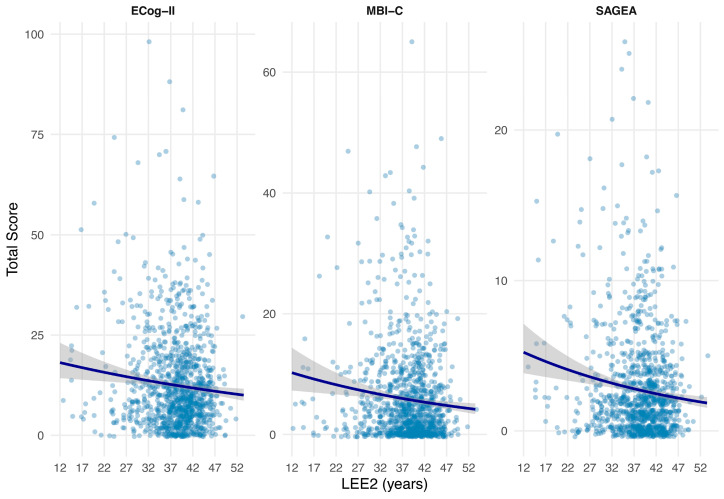
L_EE2_ associations with cognition, behavior, and function. Negative binomial regression models used to assess associations of L_EE2_ (scaled in 5-year increments) with ECog-II, MBI-C, and SAGEA scores. Shaded regions represent 95% confidence intervals. Abbreviations: ECog-II, Revised Everyday Cognition scale; MBI-C, Mild Behavioral Impairment Checklist; SAGEA, Standard Assessment of Global Everyday Activities scale.

L_EE2_ was neither associated with global or domain-specific neuropsychological performance (Table 2), nor with ECog-II score (CR = 0.95, 95% CI [0.90, 1.01], *p* = 0.101) (Figure 2).

### 3.2. L_EE2_ and Behavior

L_EE2_ was associated with severity of MBI symptoms (Figure 2). Each additional five years of L_EE2_ corresponded to an estimated 11.0% lower expected MBI-C total score (CR = 0.89, 95% CI [0.82, 0.97], *p* = 0.006).

### 3.3. L_EE2_ and Function

L_EE2_ was associated with severity of subjective functional impairment (Figure 2). Each additional five years of L_EE2_ corresponded to an estimated 9.0% lower SAGEA total score (CR = 0.91, 95% CI [0.84, 0.98], *p* = 0.010).

### 3.4. MHT Use

MHT ever use was not associated with subjective cognitive, behavioral, or functional outcomes (Table 3). Similarly, no associations were observed between MHT use and all neuropsychological measures.

## 4. Discussion

Among 1156 postmenopausal females without a diagnosis of dementia, longer L_EE2_ was associated with lower severity of behavioral and functional symptoms. No statistically significant associations were observed between L_EE2_ and objective or subjective cognitive outcomes.

In the present study, L_EE2_ associations with all measures of cognition were not statistically significant. Although longer L_EE2_ has been proposed to confer neuroprotective effects on cognition, findings across studies remain mixed [44,45] and are further compounded by limited investigation. Some studies report that among cognitively unimpaired females, longer reproductive spans are associated with reduced subjective cognitive complaints [15,36], lower white matter hyperintensity burden [14], and better memory [12] and verbal fluency [46]. Differences between our findings and prior work may reflect variation in how L_EE2_ is operationalized. While our measure of L_EE2_ incorporated reproductive span and parity, other studies account for factors such as breastfeeding [15,36], a factor not captured in CAN-PROTECT. Notably, studies incorporating breastfeeding [15,36] into L_EE2_ calculations have reported positive associations with cognition, while studies limited to reproductive span alone have not consistently observed such relationships [44]. These findings suggest that L_EE2_ should be interpreted as an approximation of E2 exposure, and differences in its construction may contribute to variability in observed associations. Additionally, our participants were highly educated (mean of 15.8 years of schooling), and greater years of education are associated with higher cognitive reserve [47,48], an established resilience factor against cognitive decline and dementia. Higher educational attainment may contribute to attenuating detectable associations between L_EE2_ and cognitive performance and may also reflect broader socioeconomic differences not captured in the present study.

Extending beyond cognition, the present findings correlate longer L_EE2_ to lower severity of subjective behavioral and functional complaints, markers that have been largely overlooked in understanding dementia risk early in the disease course. Although limited, preliminary work has shown that earlier menopause [28] and shorter L_EE2_ [49,50] is associated with more depressive symptoms in later life. In parallel, a systematic review suggests that among postmenopausal females, spontaneous premature menopause may associate with later-life physical functional decline [29], including lower gait speed and grip strength. Our findings further align with prior work showing that greater menopause symptom burden [51], which could reflect the intensity of hormonal fluctuations during the menopause transition [52], is correlated with more severe MBI symptoms. These results collectively suggest that both the timing and duration of estrogenic exposure may relate to later-life brain health. Although functional symptoms were not examined in the prior work, evidence links menopause to greater functional difficulties in later life [53,54], potentially due to long-term impact of E2 loss on neural [55], vascular [56], and skeletal [57,58] systems, independent of age. Thus, lower E2 exposure—whether from earlier menopause or shorter L_EE2_ may—associate with greater behavioral and functional complaints. In comparison, prolonged exposure may contribute to preserving brain integrity through its effects on vascular health [8], synaptic plasticity [9], neurotransmitter regulation [10], inflammation [11], and the clearance of AD pathological proteins [7,55]. These mechanisms may underpin behavioral and functional resilience, analogous to the protective effects of greater years of education or factors that promote higher cognitive reserve [47,48]. Collectively, these findings expand current evidence by emphasizing that correlations between L_EE2_ and AD risk may manifest not only through cognitive pathways, but also through behavioral and functional changes that are equally relevant to dementia risk in females.

In contrast to our L_EE2_ findings, MHT use was neither associated with objective and subjective cognitive measures, nor with subjective behavioral or functional outcomes. However, MHT use trended toward better verbal reasoning performance. Some prior studies have reported greater processing speed among MHT users compared to non-users [59,60], although findings remain inconsistent. Variability across studies may reflect differences in MHT duration and formulation, as well as the timing of MHT initiation, which was not directly examined in the present study. Moreover, these results align with the broader literature demonstrating inconsistent associations between MHT use and later-life brain outcomes [30,47,61]. For example, while longer L_EE2_ has been linked to lower white matter hyperintensity burden, no comparable association was observed for exogenous E2 use [14], which comprised of both MHT and hormonal contraceptives used in earlier life. Conversely, another study [62] found that birth control pill and MHT use following premature oophorectomy associated with better episodic memory and visuospatial processing in later life. Similar findings have been noted among females who experienced spontaneous menopause at older ages [63] but these studies only explored age at menopause and not L_EE2_ duration, which may influence outcomes. Importantly, our primary analyses classified MHT use as never versus ever use, an approach that may obscure potential differences based on recency of use. To explore whether correlations differed according to MHT status (never, past, or current use), we conducted post hoc sensitivity analyses. Although MHT status was not directly assessed in CAN-PROTECT, we derived status using reported age at initiation and duration of use to estimate the end age relative to participants’ current age. In these exploratory models, current MHT use was independently associated with better global neuropsychological performance (*p* = 0.015) and verbal reasoning (*p* < 0.001) compared to non-users, whereas past use was not associated with cognitive performance. No associations were observed between MHT status and behavioral and functional outcomes. These findings raise the possibility that correlations between MHT and cognition may depend on recency of exposure rather than lifetime history of use alone.

Nevertheless, given the cross-sectional design and post hoc nature of these analyses, these results should be interpreted cautiously. Furthermore, longitudinal investigations incorporating detailed characterization of MHT duration, formulation, and timing of initiation, in addition to MHT status, should be conducted to clarify relationships.

Several limitations to the current study warrant consideration. First, the cross-sectional design precludes inference about temporal relationships between L_EE2_ and later-life dementia risk markers. Longitudinal data are needed to clarify whether the modest effects observed predict future decline, as even small differences may be meaningful in a largely unimpaired sample. Second, our L_EE2_ measure represents a simplified proxy of cumulative endogenous E2 exposure and does not capture important contributors to hormonal variability, including breastfeeding history, menstrual cycle characteristics, or pregnancy-related factors such as trimester-specific changes and complications. The incorporation of pregnancy as a fixed duration also assumes uniform exposure, which does not reflect biological variability. These unmeasured factors may influence observed associations. Third, MHT use was categorical and lacked critical details about timing of initiation, duration, dosage, or formulation—factors that may modify the study outcomes [30,64]. Relatedly, information on hormonal contraceptive use (i.e., birth control pills) during reproductive years was unavailable, despite widespread use [65] of estrogen-containing birth control pills that may alter L_EE2_. Fourth, several important variables were not adjusted for in our analyses that may influence results. Apolipoprotein E genotype, a well-established genetic risk factor for AD that may also modify associations between E2 and brain health [66], was not assessed. Socioeconomic status, which may influence both reproductive history and dementia risk [67], was also unavailable. In addition, other factors—including vascular risk profiles, psychiatric history, and other medication use—were not comprehensively captured. The absence of these variables introduces the possibility of residual confounding, which may bias observed correlations. Finally, recall bias in self-reported reproductive history may affect L_EE2_ accuracy. This limitation extends to much of the CAN-PROTECT dataset, which relies on retrospective self-reporting. Although mean reported ages at menarche and menopause were consistent with global averages [68], recall error is possible, particularly in older participants. Such error is likely non-differential with respect to outcomes and may bias associations toward the null. Similarly, while instruments such as the ECog-II, MBI-C, and SAGEA are validated and clinically informative of AD risk, subjective reports may be influenced by individual perception or reporting tendencies.

Additionally, the CAN-PROTECT cohort is characterized by relatively high educational attainment and predominately European ethnocultural backgrounds. As such, findings may not generalize to populations with lower educational attainment or more diverse socioeconomic and ethnocultural profiles. Future studies should aim to examine these findings using biomarker and hormonal data in diverse cohorts, and extend findings to domain-level cognitive, behavioral, and functional outcomes to reveal more nuanced associations not captured in global scores.

Despite these limitations, the study offers several notable strengths. Leveraging a large, well-characterized, population-based Canadian cohort enhances the generalizability of findings. Our operationalization of L_EE2_ includes both reproductive duration and pregnancies, providing a more comprehensive estimate of cumulative endogenous E2 exposure. Prior studies often consider only reproductive duration, overlooking pregnancies, an experience that can substantially increase E2 levels [13], and may independently associate with dementia risk [69,70] and resilience [71]. Finally, by simultaneously examining cognition, behavior, and function, this study expands the scope of female brain aging research and supports a multidimensional approach to identifying early indicators of dementia risk.

## 5. Conclusions

We provide evidence from a cross-sectional study of dementia-free postmenopausal females that longer L_EE2_ correlates with lower severity of subjective behavioral and functional complaints. These results underscore the potential contributing role of L_EE2_ in supporting later-life brain health. Furthermore, these findings highlight the importance of expanding female dementia risk identification beyond cognition to include behavioral and functional changes.

## Figures and Tables

**Table 1:** Participant demographics.

Variable	M (SD), Range	*N* (%)
Age (years)	63.9 (7.4), 43.0–88.0	---
Education (years)	15.8 (4.5), 1.0–30.0	---
Ethnocultural background		
European origins	---	984 (85.1)
Non-European origins	---	172 (14.9)
Menarche age (years)	12.7 (1.5), 8.0–23.0	---
Menopause age (years)	49.7 (5.8), 21.0–65.0	---
Menopause type		
Spontaneous	---	878 (76.0)
Surgical	---	186 (16.1)
Other reasons	---	92 (8.0)
Biological children (number)		
0 children	---	268 (23.2)
1 child	---	160 (13.8)
2 children	---	471 (40.7)
3 children	---	205 (17.7)
4 children	---	44 (3.8)
5 children	---	5 (0.4)
6+ children	---	3 (0.3)
Time pregnant (years)	1.3 (0.9), 0–4.5	---
Age at first childbirth	27.3 (5.2), 14.0–42.0	---
BMI	24.3 (4.8), 11.4–77.6	
L_EE2_ (years)	38.2 (5.9), 12.0–53.5	
MHT ever use	---	406 (35.1)
Neuropsychological global score	0 (0.5), −1.2–1.7	---
ECog-II score	12.5 (11.8), 0.0–98.0	---
MBI-C score	5.9 (7.7), 0.0–65.0	---
SAGE score	2.7 (3.5), 0.0–26.0	---

Participant demographics. Mean (M), standard deviation (SD), and ranges were calculated for continuous variables. Total number (*N*) and percentage (%) were calculated for categorical variables. Abbreviations: BMI, body mass index; L_EE2,_ lifetime endogenous estradiol exposure; MHT, menopause hormone therapy; ECog-II, Revised Everyday Cognition scale; MBI-C, Mild Behavioral Impairment Checklist; SAGEA, Standard Assessment of Global Everyday Activities scale.

**Table 2:** Neuropsychological performance.

Variable	b Coefficient	95% CI [2.5, 97.5]	*p* Value
Global score	0.00	[−0.02, 0.03]	0.792
Trail Making B	0.01	[−0.02, 0.05]	0.366
Switching Stroop	0.04	[−0.01, 0.10]	0.090
Self-Ordered Search	0.02	[−0.03, 0.08]	0.380
Paired Associate Learning	−0.04	[−0.09, 0.01]	0.091
Verbal Reasoning	−0.01	[−0.06, 0.04]	0.704
Digit Span	−0.01	[−0.05, 0.03]	0.698

Association of L_EE2_ (scaled in 5-year increments) with neuropsychological performance. Coefficient estimates are presented as standardized unit differences for neuropsychological domain performances. Estimates are adjusted for MHT ever use, menopause type (spontaneous, surgical, other), age at first childbirth, BMI, age, years of education, and ethnocultural (European vs no European) background. Abbreviations: MHT, menopause hormone therapy; BMI, body mass index.

**Table 3:** MHT use.

Variable	CR	b	95% CI [2.5, 97.5]	*p*-Value
Global neuropsychological score	---	0.05	[−0.02, 0.09]	0.161
Trail Making B	---	0.01	[−0.07, 0.05]	0.780
Switching Stroop	---	0.05	[−0.05, 0.14]	0.376
Self-Ordered Search	---	0.02	[−0.10, 0.11]	0.918
Paired Associate Learning	---	0.04	[−0.07, 0.14]	0.489
Verbal Reasoning	---	0.12	[0.00, 0.23]	0.053
Digit Span	---	0.05	[−0.03, 0.14]	0.230
ECog-II total score	1.01	---	[0.89, 1.15]	0.876
MBI-C total score	0.96	---	[0.81, 1.15]	0.685
SAGEA total score	1.02	---	[0.87, 1.20]	0.793

Exploratory associations of MHT ever use with neuropsychological performance, ECog-II, MBI-C, and SAGEA scores. Coefficient estimates are presented as standardized unit differences for neuropsychological domain performances and count ratios for ECog-II, MBI-C, and SAGEA scores. Estimates are adjusted for L_EE2_, menopause type (spontaneous, surgical, other), age at first childbirth, BMI, age, years of education, and ethnocultural (European vs no European) background. Abbreviations: MHT, menopause hormone therapy; ECog-II, Revised Everyday Cognition scale; MBI-C, Mild Behavioral Impairment Checklist; SAGEA, Standard Assessment of Global Everyday Activities scale; BMI, body mass index.

## Data Availability

The data presented in this study are available on request from the corresponding author due to ethical and legal reasons.

## Abbreviations

The following abbreviations are used in this manuscript:

AD	Alzheimer disease
E2	Estradiol
L_EE2_	Lifetime exposure to endogenous estradiol
NPS	Neuropsychiatric symptoms
MBI	Mild behavioral impairment
MHT	Menopause hormone therapy
CAN-PROTECT	Canadian Platform for Research Online to Investigate Health, Quality of Life, Cognition, Behaviour, Function, and Caregiving in Aging
ECog-II	Revised Everyday Cognition scale
MBI-C	Mild Behavioral Impairment Checklist
SAGEA	Standard Assessment of Global Everyday Activities
CR	Count ratio
BMI	Body mass index

## References

[1] Frisoni G.B., Hansson O., Nichols E., Garibotto V., Schindler S.E., van der Flier W.M., Jessen F., Villain N., Arenaza-Urquijo E.M., Crivelli L. (2025). New landscape of the diagnosis of Alzheimer’s disease. Lancet.

[2] Nichols E., Steinmetz J.D., Vollset S.E., Fukutaki K., Chalek J., Abd-Allah F., Abdoli A., Abualhasan A., Abu-Gharbieh E., Akram T.T. (2022). Estimation of the global prevalence of dementia in 2019 and forecasted prevalence in 2050: An analysis for the Global Burden of Disease Study 2019. Lancet Public Health.

[3] O’Neal M.A. (2024). Women and the risk of Alzheimer’s disease. Front. Glob. Women’s Health.

[4] Castro-Aldrete L., Einsiedler M., Novakova Martinkova J., Depypere H., Alvin Ang T.F., Mielke M.M., Sindi S., Eyre H.A., Au R., Schumacher Dimech A.M. (2025). Alzheimer disease seen through the lens of sex and gender. Nat. Rev. Neurol..

[5] Zhang Y., Chen J., Li Y., Jiao B., Luo S. (2025). Disease-modifying therapies for Alzheimer’s disease: Clinical trial progress and opportunity. Ageing Res. Rev..

[6] Smith E.E., Phillips N.A., Feldman H.H., Borrie M., Ganesh A., Henri-Bhargava A., Desmarais P., Frank A., Badhwar A., Barlow L. (2025). Use of lecanemab and donanemab in the Canadian healthcare system: Evidence, challenges, and areas for future research. J. Prev. Alzheimer’s Dis..

[7] Wang X., Feng S., Deng Q., Wu C., Duan R., Yang L. (2024). The role of estrogen in Alzheimer’s disease pathogenesis and therapeutic potential in women. Mol. Cell. Biochem..

[8] Thurston R.C., Chang Y., Wu M., Harrison E.M., Aizenstein H.J., Derby C.A., Barinas-Mitchell E., Maki P.M. (2024). Reproductive hormones in relation to white matter hyperintensity volumes among midlife women. Alzheimer’s Dement..

[9] Smejkalova T., Woolley C.S. (2010). Estradiol acutely potentiates hippocampal excitatory synaptic transmission through a presynaptic mechanism. J. Neurosci..

[10] Bendis P.C., Zimmerman S., Onisiforou A., Zanos P., Georgiou P. (2024). The impact of estradiol on serotonin, glutamate, and dopamine systems. Front. Neurosci..

[11] Au A., Feher A., McPhee L., Jessa A., Oh S., Einstein G. (2016). Estrogens, inflammation and cognition. Front. Neuroendocrinol..

[12] Oughli H.A., Nguyen S.A., Siddarth P., Fox M., Milillo M., Ercoli L., Lavretsky H. (2022). The effect of cumulative lifetime estrogen exposure on cognition in depressed versus non-depressed older women. J. Geriatr. Psychiatry Neurol..

[13] Dukic J., Johann A., Henninger M., Ehlert U. (2024). Estradiol and progesterone from pregnancy to postpartum: A longitudinal latent class analysis. Front. Glob. Women’s Health.

[14] Cote S., Perron T.-L., Baillargeon J.-P., Bocti C., Lepage J.-F., Whittingstall K. (2023). Association of cumulative lifetime exposure to female hormones with cerebral small vessel disease in postmenopausal women in the UK biobank. Neurology.

[15] Matyi J.M., Rattinger G.B., Schwartz S., Buhusi M., Tschanz J.T. (2019). Lifetime estrogen exposure and cognition in late life: The Cache County Study. Menopause.

[16] Park H.K., Marston L., Mukadam N. (2024). The effects of estrogen on the risk of developing dementia: A cohort study using the UK biobank data. Am. J. Geriatr. Psychiatry.

[17] Huang T., Shafrir A.L., Eliassen A.H., Rexrode K.M., Tworoger S.S. (2019). Estimated Number of Lifetime Ovulatory Years and Its Determinants in Relation to Levels of Circulating Inflammatory Biomarkers. Am. J. Epidemiol..

[18] Borda M.G., Aarsland D., Tovar-Rios D.A., Giil L.M., Ballard C., Gonzalez M.C., Brønnick K., Alves G., Oppedal K., Soennesyn H. (2020). Neuropsychiatric symptoms and functional decline in Alzheimerʼs disease and Lewy body dementia. J. Am. Geriatr. Soc..

[19] Ismail Z., Smith E.E., Geda Y., Sultzer D., Brodaty H., Smith G., Agüera-Ortiz L., Sweet R., Miller D., Lyketsos C.G. (2016). Neuropsychiatric symptoms as early manifestations of emergent dementia: Provisional diagnostic criteria for mild behavioral impairment. Alzheimer’s Dement..

[20] Guan D.X., Rehman T., Nathan S., Durrani R., Potvin O., Duchesne S., Pike G.B., Smith E.E., Ismail Z. (2024). Neuropsychiatric symptoms: Risk factor or disease marker? A study of structural imaging biomarkers of Alzheimer’s disease and incident cognitive decline. Hum. Brain Mapp..

[21] Ismail Z., Leon R., Creese B., Ballard C., Robert P., Smith E.E. (2023). Optimizing detection of Alzheimer’s disease in mild cognitive impairment: A 4-year biomarker study of mild behavioral impairment in ADNI and MEMENTO. Mol. Neurodegener..

[22] Leon R., Ghahremani M., Guan D.X., Smith E.E., Zetterberg H., Ismail Z. (2026). Enhancing Alzheimer Disease Detection Using Neuropsychiatric Symptoms: The Role of Mild Behavioural Impairment in the Revised NIA-AA Research Framework. J. Geriatr. Psychiatry Neurol..

[23] Ghahremani M., Leon R., Smith E.E., Ismail Z. (2025). Exploring the association between mild behavioral impairment and plasma p-tau217: Implications for early detection of Alzheimer’s disease. Alzheimer’s Dement. Diagn. Assess. Dis. Monit..

[24] Ghahremani M., Smith E.E., Ismail Z. (2026). Persistent functional impairment as an early indicator of cognitive decline and dementia in cognitively normal older adults. J. Alzheimers Dis..

[25] Ghahremani M., Smith E.E., Ismail Z. (2025). Persistent Functional Impairment as an Early Indicator of Alzheimer Disease Pathology and Progression. J. Am. Geriatr. Soc..

[26] Vassilaki M., Aakre J.A., Kremers W.K., Mielke M.M., Geda Y.E., Machulda M.M., Knopman D.S., Coloma P.M., Schauble B., Vemuri P. (2018). Association between functional performance and Alzheimer’s disease biomarkers in individuals without dementia. J. Am. Geriatr. Soc..

[27] Arruda F., Rosselli M., Greig M.T., Loewenstein D.A., Lang M., Torres V.L., Vélez-Uribe I., Conniff J., Barker W.W., Curiel R.E. (2021). The association between functional assessment and structural brain biomarkers in an ethnically diverse sample with normal cognition, mild cognitive impairment, or dementia. Arch. Clin. Neuropsychol..

[28] van Zwol-Janssens C., Louwers Y.V., Laven J.S., Schipper J., Jiskoot G. (2025). Depressive symptoms in women with premature ovarian insufficiency (POI): A cross-sectional observational study. Menopause.

[29] de Souza Macêdo P.R., Rocha T.N., Gomes Fernandes S.G., Apolinário Vieira M.C., Jerez-Roig J., Aires da Câmara S.M. (2021). Possible association of early menopause with worse physical function: A systematic review. Menopause.

[30] Nerattini M., Jett S., Andy C., Carlton C., Zarate C., Boneu C., Battista M., Pahlajani S., Loeb-Zeitlin S., Havryulik Y. (2023). Systematic review and meta-analysis of the effects of menopause hormone therapy on risk of Alzheimer’s disease and dementia. Front. Aging Neurosci..

[31] Rocca W.A., Kantarci K., Faubion S.S. (2024). Risks and benefits of hormone therapy after menopause for cognitive decline and dementia: A conceptual review. Maturitas.

[32] Ismail Z., Guan D.X., Vellone D., Ballard C., Creese B., Corbett A., Pickering E., Bloomfield A., Hampshire A., Sekhon R. (2024). The Canadian platform for research online to investigate health, quality of life, cognition, behaviour, function, and caregiving in aging (CAN-PROTECT): Study protocol, platform description, and preliminary analyses. Aging Health Res..

[33] Guan D.X., Aundhakar A., Tomaszewski Farias S., Ballard C., Creese B., Corbett A., Pickering E., Roach P., Smith E.E., Ismail Z. (2025). Vascular risk factor associations with subjective cognitive decline and mild behavioural impairment. Brain Commun..

[34] Guan D.X., Peters M.E., Pike G.B., Ballard C., Creese B., Corbett A., Pickering E., Roach P., Smith E.E., Ismail Z. (2025). Cognitive, behavioral, and functional outcomes of suspected mild traumatic brain injury in community-dwelling older persons without mild cognitive impairment or dementia. J. Acad. Consult.-Liaison Psychiatry.

[35] Mudalige D., Guan D.X., Ballard C., Creese B., Corbett A., Pickering E., Roach P., Smith E.E., Ismail Z. (2024). The mind and motion: Exploring the interplay between physical activity and Mild Behavioral Impairment in dementia-free older adults. Int. Rev. Psychiatry.

[36] Fox M., Berzuini C., Knapp L.A. (2013). Cumulative estrogen exposure, number of menstrual cycles, and Alzheimer’s risk in a cohort of British women. Psychoneuroendocrinology.

[37] Nelson L.R., Bulun S.E. (2001). Estrogen production and action. J. Am. Acad. Dermatol..

[38] Brooker H., Williams G., Hampshire A., Corbett A., Aarsland D., Cummings J., Molinuevo J.L., Atri A., Ismail Z., Creese B. (2020). FLAME: A computerized neuropsychological composite for trials in early dementia. Alzheimer’s Dement. Diagn. Assess. Dis. Monit..

[39] Farias S.T., Weakley A., Harvey D., Chandler J., Huss O., Mungas D. (2021). The measurement of Everyday Cognition (ECog): Revisions and updates. Alzheimer Dis. Assoc. Disord..

[40] Ismail Z., Agüera-Ortiz L., Brodaty H., Cieslak A., Cummings J., Fischer C.E., Gauthier S., Geda Y.E., Herrmann N., Kanji J. (2017). The Mild Behavioral Impairment Checklist (MBI-C): A rating scale for neuropsychiatric symptoms in pre-dementia populations. J. Alzheimer’s Dis..

[41] Ismail Z., McGirr A., Gill S., Hu S., Forkert N.D., Smith E.E. (2021). Mild behavioral impairment and subjective cognitive decline predict cognitive and functional decline. J. Alzheimer’s Dis..

[42] Marzona I. (2011). The Standard Assessment of Global Activities in the Elderly (SAGE) Scale: Validation Process of a New Tool for the Assessment of Disability in Older Adults. Ph.D. Thesis.

[43] Phelps J., Guan D.X., Teo K., O’Donnell M., Yusuf S., Bosch J., Ismail Z., Smith E.E. (2025). Validation of the standard assessment of global everyday activities (SAGEA) scale for dementia diagnosis. Age Ageing.

[44] Low L.-F., Anstey K., Jorm A., Rodgers B., Christensen H. (2005). Reproductive period and cognitive function in a representative sample of naturally postmenopausal women aged 60–64 years. Climacteric.

[45] Georgakis M.K., Kalogirou E.I., Diamantaras A.-A., Daskalopoulou S.S., Munro C.A., Lyketsos C.G., Skalkidou A., Petridou E.T. (2016). Age at menopause and duration of reproductive period in association with dementia and cognitive function: A systematic review and meta-analysis. Psychoneuroendocrinology.

[46] Ryan J., Carrière I., Scali J., Ritchie K., Ancelin M.-L. (2009). Life-time estrogen exposure and cognitive functioning in later life. Psychoneuroendocrinology.

[47] Jett S., Malviya N., Schelbaum E., Jang G., Jahan E., Clancy K., Hristov H., Pahlajani S., Niotis K., Loeb-Zeitlin S. (2022). Endogenous and exogenous estrogen exposures: How women’s reproductive health can drive brain aging and inform Alzheimer’s prevention. Front. Aging Neurosci..

[48] Guan D.X., Mortby M.E., Pike G.B., Ballard C., Creese B., Corbett A., Pickering E., Hampshire A., Roach P., Smith E.E. (2024). Linking cognitive and behavioral reserve: Evidence from the CAN-PROTECT study. Alzheimer’s Dement..

[49] Wu Q., Yan Y., La R., Zhang X., Lu L., Xie R., Xue Y., Lin C., Xu W., Xu J. (2024). Association of reproductive lifespan and age at menopause with depression: Data from NHANES 2005–2018. J. Affect. Disord..

[50] Georgakis M.K., Thomopoulos T.P., Diamantaras A.-A., Kalogirou E.I., Skalkidou A., Daskalopoulou S.S., Petridou E.T. (2016). Association of age at menopause and duration of reproductive period with depression after menopause: A systematic review and meta-analysis. JAMA Psychiatry.

[51] Crockford J.F., Guan D.X., Einstein G., Ballard C., Creese B., Corbett A., Pickering E., Bloomfield A., Roach P., Smith E.E. (2025). Menopausal symptom burden as a predictor of mid-to late-life cognitive function and mild behavioral impairment symptoms: A CAN-PROTECT study. PLoS ONE.

[52] Al-Azzawi F., Palacios S. (2009). Hormonal changes during menopause. Maturitas.

[53] Tseng L.A., El Khoudary S.R., Young E.A., Farhat G.N., Sowers M., Sutton-Tyrrell K., Newman A.B. (2012). The association of menopause status with physical function: The Study of Women’s Health Across the Nation. Menopause.

[54] Sowers M., Tomey K., Jannausch M., Eyvazzadeh A., Nan B., Randolph J. (2007). Physical functioning and menopause states. Obstet. Gynecol..

[55] Mosconi L., Berti V., Dyke J., Schelbaum E., Jett S., Loughlin L., Jang G., Rahman A., Hristov H., Pahlajani S. (2021). Menopause impacts human brain structure, connectivity, energy metabolism, and amyloid-beta deposition. Sci. Rep..

[56] Aittokallio J., Saaresranta T., Riskumäki M., Hautajärvi T., Vahlberg T., Polo O., Heinonen O., Raitakari O., Kalleinen N. (2023). Effect of menopause and age on vascular impairment. Maturitas.

[57] Karlamangla A.S., Burnett-Bowie S.-A.M., Crandall C.J. (2018). Bone health during the menopause transition and beyond. Obstet. Gynecol. Clin. N. Am..

[58] Wright V.J., Schwartzman J.D., Itinoche R., Wittstein J. (2024). The musculoskeletal syndrome of menopause. Climacteric.

[59] Greendale G.A., Huang M.H., Wight R.G., Seeman T., Luetters C., Avis N.E., Johnston J., Karlamangla A.S. (2009). Effects of the menopause transition and hormone use on cognitive performance in midlife women. Neurology.

[60] Calvo N., Einstein G. (2023). Steroid hormones: Risk and resilience in women’s Alzheimer disease. Front. Aging Neurosci..

[61] Lee J.K., Frank R.D., Christenson L.R., Fields J.A., Rocca W.A., Mielke M.M. (2024). Associations of reproductive factors and exogenous estrogens with global and domain-specific cognition in later life. Alzheimer’s Dement..

[62] Watts A., Donofry S., Ripperger H., Eklund N.M., Wan L., Kang C., Grove G., Oberlin L.E., Gujral S., Vidoni E.D. (2025). Lifetime estrogen exposure and domain-specific cognitive performance: Results from the IGNITE study. Front. Aging Neurosci..

[63] Puri T.A., Gravelsins L.L., Alexander M.W., McGovern A.J., Guterman P.D., Rabin J.S., Murphy K.J., Galea L.A. (2025). Association between menopause age and estradiol-based hormone therapy with cognitive performance in cognitively normal women in the CLSA. Neurology.

[64] Mosconi L., Andy C., Nerattini M., Ajila T., Zarate C., Boneu C., Fauci F., Battista M., Pahlajani S., Christos P. (2025). Systematic Review and Meta-analysis of Menopause Hormone Therapy (MHT) and the Risk of Alzheimer’s Disease and All-cause Dementia: Effects of MHT Characteristics, Location, and APOE-4 Status. Curr. Obstet. Gynecol. Rep..

[65] Campbell A.J., Claydon V.E., Liva S., Cote A.T. (2025). Changes in Canadian contraceptive choices: Results of a national survey on hormonal contraceptive use. BMC Women’s Health.

[66] Altmann A., Tian L., Henderson V.W., Greicius M.D. (2014). Alzheimer’s Disease Neuroimaging Initiative Investigators. Sex modifies the APOE-related risk of developing Alzheimer disease. Ann. Neurol..

[67] Wang A.Y., Hu H.Y., Ou Y.N., Wang Z.T., Ma Y.H., Tan L., Yu J.T. (2023). Socioeconomic Status and Risks of Cognitive Impairment and Dementia: A Systematic Review and Meta-Analysis of 39 Prospective Studies. J. Prev. Alzheimers Dis..

[68] Bazyar N., Moradi Z., Khani Jeihooni A., Dehghan A. (2025). Trends in age at natural menopause and menarche and related factors in Iran: Results from a population-based study. Sci. Rep..

[69] Bae J., Lipnicki D., Han J., Kim T.H., Sachdev P.S., Kwak K.P., Kim B.J., Kim S.G., Kim J.L., Moon S.W. (2020). Parity and the risk of incident dementia: A COSMIC study. Epidemiol. Psychiatr. Sci..

[70] Bae J.B., Lipnicki D.M., Han J.W., Sachdev P.S., Kim T.H., Kwak K.P., Kim B.J., Kim S.G., Kim J.L., Moon S.W. (2020). Does parity matter in women’s risk of dementia? A COSMIC collaboration cohort study. BMC Med..

[71] Fu C., Hao W., Ma Y., Shrestha N., Virani S.S., Mishra S.R., Zhu D. (2023). Number of live births, age at the time of having a child, span of births and risk of dementia: A population-based cohort study of 253,611 UK women. J. Women’s Health.

